# Prevalence and incidence of personality disorders among children and adolescents in Danish mental health services: a nationwide register study

**DOI:** 10.1007/s00787-023-02274-w

**Published:** 2023-08-11

**Authors:** Ida Maria Ingeholm Klinkby, Lene Halling Hastrup, Sune Bo, Ole Jakob Storebø, Erik Simonsen, Mickey T. Kongerslev

**Affiliations:** 1 Psychiatric Research Unit, Mental Health Services Region Zealand, Slagelse, Denmark; 2 Department of Psychology, University of Copenhagen, Copenhagen, Denmark; 3 Department of Psychology, University of Southern Denmark, Odense, Denmark; 4 Research Unit, Region Zealand Mental Health Services East, Roskilde, Denmark; 5 Department of Clinical Medicine, University of Copenhagen, Copenhagen, Denmark; 6 Danish Centre for Health Economics (DaCHE), University of Southern Denmark, Odense, Denmark

**Keywords:** Borderline personality disorder, Childhood, Adolescence, Mental health service, Epidemiology, Service use

## Abstract

A few epidemiological studies have examined personality disorders (PDs) among children and adolescents in secondary mental health services. This study aims to describe the prevalence and incidence of PDs among children and adolescents who have attended Danish child and adolescent psychiatric services (CAPS). Using register-based data, we studied all patients under the age of 18 years who were admitted to in- and outpatient CAPS (*N* = 115,121) in Denmark from 2007 to 2017. A total of 4952 patients were diagnosed with a PD during the study period. The mean prevalence was 859 patients per year, and the mean incidence was 274 patients per year, including an increased incidence and prevalence of borderline, anxious, and unspecified PDs over the decade. The number of patients diagnosed with PDs increased from 700 to 851 per year, but the proportion of patients with PDs compared to all psychiatric diagnoses decreased from 4.2% to 2.8% over the study period. The PD population had an older age (14.8 years vs. 11.3 years; *p* < 0.001), a higher likelihood of being female (74% vs. 44%; *p* < 0.001), and four times more contacts with the psychiatric emergency departments than other patients with a psychiatric diagnosis. Future studies should focus on (a) implementing further epidemiological studies in different countries; (b) tracking diagnostic practices to facilitate comparisons and provide feedback for training clinicians and raising awareness; and (c) estimating trajectories of PDs, including costs within the CAPS, to facilitate informed decision-making regarding the future organization and provision of services to these children, adolescents, and their families.

**Supplementary Information:**

The online version contains supplementary material available at 10.1007/s00787-023-02274-w.

## Introduction

Personality disorders (PDs) are severe mental disorders that are characterized by inflexible, maladaptive, and pervasive patterns of cognition, affect, impulse regulation, and interpersonal behaviors [1, 2, 41]. In addition to this general diagnostic definition, both of the international classification systems (ICD-10 and DSM-IV/5) include criteria to specify which specific PDs an individual may meet criteria for. Whereas PDs have gained considerable recognition as severe mental disorders that significantly contribute to burdens in terms of mental and physical health [20, 36, 45, 53], functioning [24, 27], and societal costs [25] among adult populations in recent decades, their applicability in child and adolescent mental health has remained controversial [9, 10, 49]. However, over the past 2 decades, researchers and clinicians have increasingly established that PD diagnoses are no less reliable or valid in adolescents than in adults [33, 38, 48]. Indeed, empirical evidence is accumulating to suggest that PDs are developmental disorders and are thus important to diagnose, recognize, and treat in childhood and adolescence, although the current evidence is primarily based on research in adolescence, whereas knowledge of PDs in childhood still lacks a solid empirical foundation. With this caveat in mind, the available research suggests that PDs actually tend to have their peak prevalence and typical onset in early adolescence or emerging adulthood [8, 12, 29, 38]. Importantly, a growing number of studies have also demonstrated that PD diagnosed in childhood or early adulthood is associated with the current and future problems, including high comorbidity with other PDs and mental disorders [16, 47], physical illness [19], self-harm [3], poor educational attainment and life satisfaction [26, 30, 55], and a heightened risk of death by suicide compared to other mental health disorders [40]. Taken together, such research findings have accumulated to suggest that a hesitancy toward diagnosing PDs in young people may increase the risk of not recognizing a severe mental health disorder and then missing the chance to provide early targeted treatment. As such, both the DSM-5 Section III and the ICD-11 have removed any references to age in their diagnostic criteria for PDs. While the removal of references to age can be viewed as positive, in terms of reflecting the accumulating research findings in adolescence, it is somewhat more problematic in childhood, where research is still missing. Moreover, none of the two major classifications systems currently provide age-specific or developmentally sensitive criteria. In effect, child and adolescent mental health clinicians are still making diagnoses based on diagnostic PD criteria modeled after an adult phenotype.

Regarding treatment, most of the available treatment research has focused on borderline PD, and herein, the evidence suggests that a number of specialized treatments, all of which are tailored to the core psychopathology features of borderline PD, are efficacious for adolescents [52]. That said, the effect sizes for adolescents appear somewhat small, and the number of trials for this population is few [52]. Thus, despite research to support diagnosis and early intervention/treatment for adolescents with PDs, clinical scepticism and lack of availability of dedicated services and treatments for children and adolescents diagnosed with PDs appears to be a widespread barrier to future progress [5, 6, 48]. To be sure, hesitancy may be warranted in childhood, where research evidence is still missing, but not in adolescence. However, little is known about the diagnosis of PDs in clinical practice in terms of prevalence and incidence among secondary child and adolescent psychiatric services (CAPS). This knowledge would allow us to further assess the extent to which clinicians are actually complying with the current research base supporting the use of PD diagnoses in CAPS. Additionally, little is known about the prevalence of PDs among children and adolescents, not only in the community but also in nationwide mental health service systems, although notable exceptions do exist. For example, Johnson et al. [29] found that the prevalence of any PD diagnosis was 14.6% at the age of 14 years, 12.9% at the age of 16 years, and 13.9% at 22 years in a community sample of 568 US adolescents. Across representative community samples and primary care settings, prevalence estimates for any PD diagnosis have ranged from 6 to 17%, with a median of 11% in childhood and adolescence [28]. These findings are comparable to or slightly higher than those reported in epidemiological studies with adults [43]. Studies on PDs in adolescent clinical samples are scarce, but the available studies suggest, as in adults, a high prevalence, with estimates ranging from 41% [18] to 64% [23]. Again, these estimates, across countries and samples, are generally comparable to estimates reported from various adult clinical samples [57]. In terms of specific PD diagnoses, it is worth noting that although most research appears to focus on borderline PD and to some extent antisocial PD, at least in adulthood, the diagnosis of PD not otherwise specified is rarely studied but appears to be the most common PD diagnosis in both adolescent and adult samples [18, 54]. Part of the explanation for PD not otherwise specified being one of the most common in adolescence, could well be the lack of developmentally appropriate diagnostic criteria.

There is limited knowledge about the prevalence and incidence of PDs in CAPS, and the generalizability of the existing results is low considering that the few studies all focus on selected samples in clinical settings. Moreover, and again affecting interpretability and generalizability, is the fact that various measures for structured assessment are used in such studies, which is not how diagnoses are made in real-world mental health systems [39]. Thus, planners, politicians, management, researchers, and educators are left without actual knowledge on the frequency of PDs within mental health systems. Therefore, knowledge based on the actual diagnostic practice and service delivery within CAPS is necessary and might complement data gained from more rigorous and academic assessments in subsamples, with national data on the CAPS level. Indeed, one can argue that clinical diagnosis may be a more relevant indicator of disorder severity and the need for treatment [39]. Knowledge gained from such studies can provide important information back to decision-makers as well as educators and clinicians to test if current clinical practice is in accordance with current evidence and if changes in organization and treatment provision are warranted.

Against this background, the present study sought to explore the prevalence and incidence of ICD-10 PD diagnoses in secondary Danish child and adolescent psychiatric services (CAPS) in the period from 2007 to 2017 using nationwide register data. In particular, we aimed to describe the following:

1. The incidence and prevalence of PDs in CAPS for all primary and secondary PD diagnoses in total and in comparison, to all mental disorders throughout the study period.
2. The incidence and prevalence of specific PDs.
3. Gender and age differences for the group of individuals diagnosed with PDs compared to all mental disorders.
4. The proportion of individuals with PD diagnoses in contact with the CAPS’s psychiatric emergency departments compared to all mental disorders
5. Potential regional differences in Denmark in terms of PD diagnoses in CAPS.

## Methods

### Study population

We studied all admissions or contacts to secondary in- and outpatient CAPS in Denmark from 2007 to 2017. All patients under the age of 18 years with a primary or secondary ICD-10 F00–99 diagnosis were included in the study.

### Procedure and register data

All data in the present study were supplied by the Danish Health Data Authority. The data were obtained from the Danish National Patient Register (NPR) in December 2018. The NPR contains information on psychiatric inpatient admissions and outpatient contacts, including contacts with psychiatric emergency departments; information on diagnoses according to ICD-10; type of referral; place and mode of treatment; and information on municipality of residence and the patients’ age and gender [34].

### Statistical approach and definitions

Data were analyzed using STATA 15.1. Admissions were defined as any in- or outpatient contact with CAPS in Denmark during the study period, with an ICD-10 F00 to F99 primary or secondary diagnosis.

PD diagnoses were as follows: schizotypal (F21), paranoid (F60.0), schizoid (F60.1), dissocial (F60.2), emotionally unstable (F60.3x—including impulsive (F60.30) and borderline (F60.31) type), histrionic (F60.4), obsessive–compulsive (F60.5), anxious (F60.6), dependent (F60.7), other specific PDs (F60.8), unspecified PD (F60.9), and mixed (F60.1). Schizotypal disorder was included as a PD in this study to facilitate comparison with studies based on the DSM system [1, 2], wherein it is classified as a PD.

We excluded patients who were given a diagnosis in the emergency department, as these contacts often do not allow for thorough diagnostic assessment. Hence, all the included patients were diagnosed in either an outpatient clinic or an inpatient facility. We analyzed the following specific types of PD diagnoses separately: F21, F60.31, F60.6, and F60.9. All other PD diagnoses were categorized into one group due to the small number of patients diagnosed with each of these PDs.

Prevalence rates were calculated by dividing the number of patients with a PD diagnosis by the number of patients with all psychiatric admissions for each year from 2007 to 2017. Incidence rates were calculated by dividing the number of first-time PD admissions by all psychiatric first-time admissions for a given year. Data on contacts with psychiatric emergency departments were only available from 2007 to 2013. We tested for differences in incidence and prevalence across gender, age, and the group of PDs compared to all psychiatric diagnoses using the Chi-square test for categorical variables and parametric or nonparametric tests for count variables. We used *p* < 0.05 as the level of significance.

### Ethics

This study was approved by the Danish Data Protection Agency (REG-102-2018). Informed consent was not required, since all the data in the register were obtained anonymously in accordance with Danish law.

## Results

### Incidence and prevalence

**Fig. 1:**
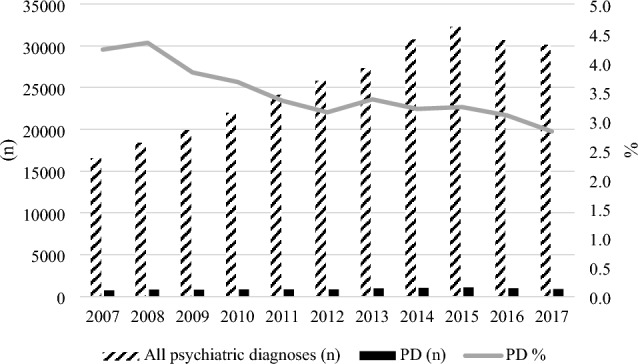
Number and proportion of PD diagnoses compared to all psychiatric diagnoses from 2007 to 2017 in secondary Danish Child and Adolescent Psychiatric Services. The figure displays the number of PD diagnoses both in terms of actual numbers (*n*) and percentages (%) on the two vertical axis to the left and right, respectively. *PD* Personality disorder

In total, 115,121 individuals were included in the study. Of these, 4952 patients were diagnosed with PD during the entire study period. From 2007 to 2017, the total number of patients in contact with CAPS in Denmark increased from 16,555 to 30,132 (see Supplemental Table S1). The yearly number of patients diagnosed with PDs increased from 700 to 851 during 2007–2017, but the proportion of patients with PD compared to all psychiatric diagnoses decreased from 4.2% to 2.8% over the study period (see Fig. 1; and Supplemental Table S1). The mean number of patients with a primary or secondary PD diagnosis over the entire study period was 859 individuals per year, which accounted for 3.4% of all psychiatric diagnoses in the same period (see Fig. 1; Supplemental Table S1). Figure 1 also illustrates that the proportion of PD patients compared to all psychiatric diagnoses decreased over the study period due to a marked increase in patients with other psychiatric diagnoses (see also Supplemental Table S1).

**Fig. 2:**
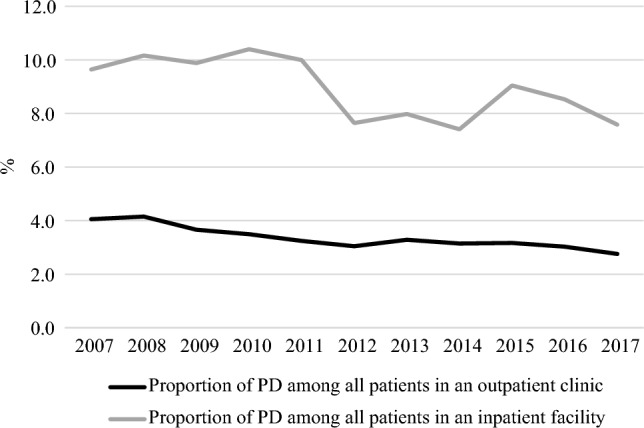
Proportion (%) of personality disorder diagnoses compared to all psychiatric diagnoses stratified by in- versus outpatient facilities in secondary Danish child and adolescent mental health services during 2007–2017. *PD* Personality disorder

In the inpatient facilities, the mean prevalence of PD was 8.9%, and in the outpatient clinics, it was 3.3% over the study period. We found that the proportion of patients with PDs was much higher in inpatient facilities than in outpatient clinics (see Fig. 2). The prevalence of any PD diagnosis varied in the inpatient facilities from 7.4 to 10.6% of all admissions and decreased over the decade from 9.6 to 7.6% (see Fig. 2). The proportion of PDs among all patients in the outpatient clinics displayed a steady decline from 4.1 to 2.8% over the decade (see Fig. 2).

**Fig. 3:**
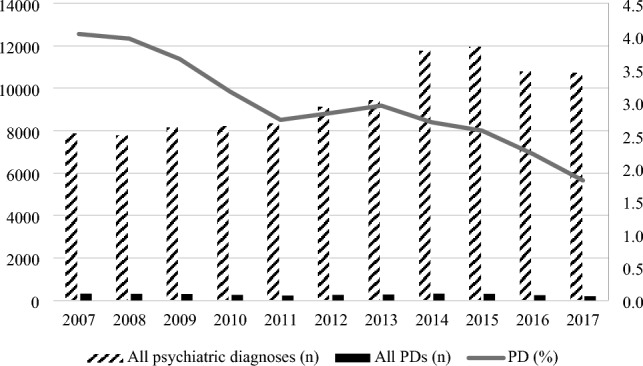
Incidence of first psychiatric admissions to secondary Danish child and adolescent psychiatric services and the proportion of personality disorder diagnoses among these from 2007 to 2017. The left vertical axis represents numbers (*n*), which the bars relate to, and the right vertical axis represents % which the line refers to. *PD*  Personality disorder

The incidence of PD decreased over the study period from 4.0% to 1.8% (see Supplemental Table S2). Regarding the specific PD types, the incidence rates of emotionally unstable PD, anxious PD, and unspecified PD increased, while the incidence rates of schizotypal and other PDs decreased over the study period (see Fig. 3).

### Prevalence of specific PDs

**Fig. 4:**
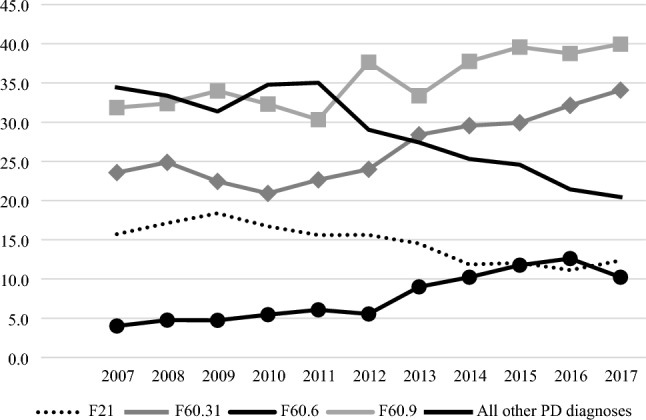
Prevalence of specific ICD-10 personality disorder diagnoses in Danish Child and Adolescent Mental Health Services from 2007 to 2017. The figure displays the percentage of various personality diagnoses compared to all personality disorder diagnoses for the study years. *PD* Personality disorder. F21: Schizotypal; F60.31: Emotionally unstable personality disorder of borderline type; F60.6: Anxious personality disorder; and F60.9 Unspecified personality disorder

Figure 4 shows the prevalence of different types of specific PDs. The prevalence rates of the following PDs increased: unspecified PD (F60.9; from 31.9% to 40.0%), anxious PD (F60.6; from 4.0% to 10.6%), and emotionally unstable, borderline type (F60.31; from 23.6% to 34.1%). The prevalence of schizotypal disorder decreased slightly (from 15.7% to 12.3%), and the prevalence of all other PD diagnoses remained almost stable from 2007 to 2011 and decreased thereafter (from 35.0% to 20.4%). Altogether, this indicates that unspecified PD was the most common diagnosis. Among the specific PDs, emotionally unstable, borderline-type PD was the most common diagnosis.

### Gender and age differences

Individuals with PD diagnoses differed significantly from the group of individuals with other psychiatric diagnoses with respect to age and gender across the study period. The age of the PD population was higher (mean = 14.8 years, SD 2.1) than that of the group with other psychiatric diagnoses (mean = 11.3 years, SD 4.3; *p* < 0.001). Only 3–6% of individuals with a PD diagnosis were under the age of 10 years, whereas more than 60% with a PD diagnosis were older than 15 years of age (see Supplemental Table S3 for details). The proportion of females in the PD population (74%) was higher than the proportion of females in the group of other psychiatric diagnoses (44%) (*p* < 0.001).

### Admissions to the psychiatric emergency departments

**Fig. 5:**
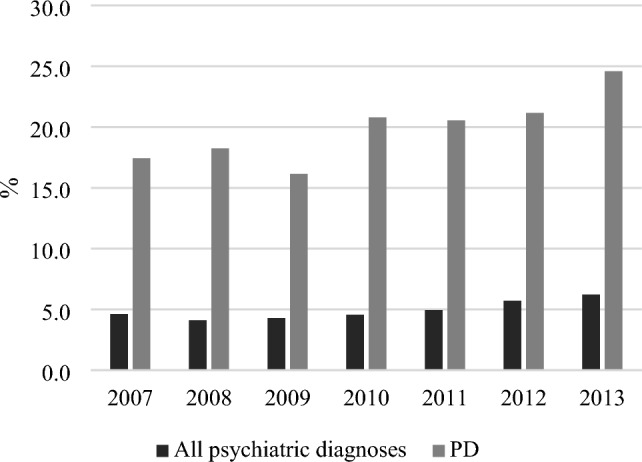
Proportion of patients with and without a personality disorder diagnosis, that had contact with Danish child and adolescent psychiatric emergency departments from 2007 to 2013. *PD*  Personality disorder.

Figure 5 shows the proportion of patients with any PD diagnosis who have been in contact with the psychiatric emergency departments within a year compared to those diagnosed with all other types of psychiatric diagnoses. The proportion of patients with contact with the psychiatric emergency departments among the PDs increased from 17.4 to 24.6% from 2007 to 2013, whereas the proportion of other psychiatric diagnoses only increased from 4.6 to 6.2%.

### Regional differences in Denmark

**Fig. 6:**
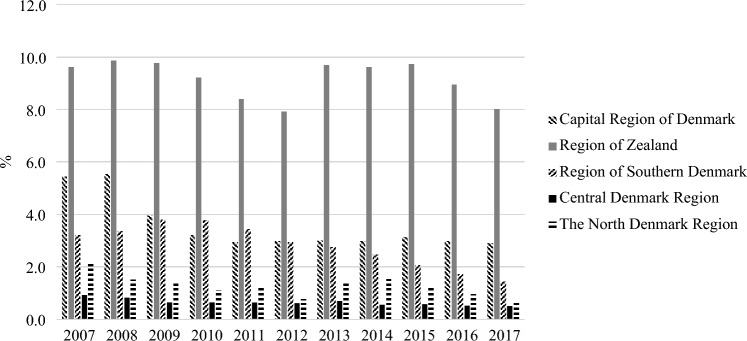
Regional differences in prevalence of personality disorder diagnoses compared to all psychiatric diagnoses in secondary Danish child and adolescent mental health services from 2007 to 2017

Finally, we explored potential regional differences in Denmark. Denmark is divided into five regions for CAPS administration. Figure 6 shows substantial differences in the prevalence of PD diagnoses across the five regions. For example, in 2017, 52.3% of all children and adolescent patients with a PD diagnosis in Denmark were in contact with the CAPS.

## Discussion

To the best of our knowledge, this is the first study to provide nationwide real-world data on the incidence and prevalence of PD diagnoses in CAPS. While the incidence and prevalence of psychiatric diagnoses among children and adolescents are relatively well described both in the community as well as in clinical settings or among specific groups (see, e.g., [14, 35, 37]), much less is known about the incidence and prevalence of PD diagnoses in children and adolescents, especially at the nationwide secondary CAPS level in the world. Thus, despite the growing recognition of the importance of assessing and treating PDs in young people among many clinicians and researchers [8], these diagnoses are often ignored in large-scale epidemiological studies [46]. This means that we lack important knowledge to make informed decisions and legislation about prevention, intervention and service delivery for this group of individuals and their significant others. The present study aimed to close some of this knowledge gap by examining the prevalence and incidence of PD diagnoses in CAPS in Denmark from 2007 to 2017.

A major finding from our study was that the total number of children and adolescents diagnosed with PD in CAPS increased from 700 to 851 over the study period, perhaps suggesting a growing recognition and awareness of PD diagnoses among clinicians. That said, the proportion of PD diagnoses in relation to all psychiatric disorders decreased from 4.2% to 2.8%. The decrease in the proportion of PD diagnoses compared with all diagnoses might partly be understood as a function of the overall 84% increase in the number of patients in contact with CAPS from 2007 to 2017. This overall dramatic increase in the number of children and adolescents in contact with CAPS is consistent with prior Danish [17] and international studies [15] and poses considerable challenges for the organization and delivery of services in the future.

The mean prevalence of PD diagnoses throughout the study period was 3.4% among the total clinical population in CAPS. In the inpatient facilities, the prevalence was 8.9%, and it was 3.3% in the outpatient clinics. Although we do not have other studies on the prevalence of PDs in inpatient settings with which to make comparisons, the prevalence in outpatient settings was somewhat lower when compared to prior international studies [33]. These findings are in accordance with our findings of the incidence of PD that decreased where all other diagnoses increased during the decade. As we are not aware of previous nationwide studies reporting on the nationwide prevalence of PD diagnoses in CAPS, we cannot directly compare our findings with other international studies.

To our knowledge, this is also the first study in Denmark looking at the prevalence and incidence of PD diagnoses in a nationwide Danish clinical population of children and adolescents. Pedersen et al. [44] found that the prevalence of PD among adults (> 18 years) in Danish adult psychiatric services from 1995 to 2006 was 15.4%. In comparison, our prevalence rates were considerably lower. Dalsgaard et al. [17] found 2631 incident cases of PD among Danish children and adolescents born between 1995 and 2016 followed from birth to December 2016. This study, furthermore, reported a cumulative prevalence of 2‰ among all children and adolescents, which corresponds to our findings. Dalsgaard et al. also found a significantly higher cumulative incidence of PD diagnosis before the age of 18 years among girls than among boys (1.05% vs. 0.3%). This also corresponds to our finding of a significantly higher rate of girls in the PD group than in the group of all other psychiatric disorders (74% vs. 44%).

Our study population of PD had a significantly higher age at the first admission compared to all psychiatric disorders, perhaps indicating that these diagnoses are still applied rather late in the developmental course [21], which again could reflect not only scepticism on behalf of the clinicians, but also the limited research on PDs in childhood, as well as developmentally sensitive diagnostic criteria. Thus, caution in using these diagnoses in children may be warranted; on the other hand, many researchers are arguing for early detection (i.e., diagnosis), as it is important for proper early intervention, which again may hold the promise of warding off further problems arising from delayed diagnosis and treatment [11, 32, 33, 48]. Our data were collected via the Danish National Patient Register and compared all Danish children and adolescents under the age of 18 years. This could partially explain the lower prevalence of PD in our study than among the other studies with a higher age minimum [33]. Many prior studies have looked at a specific population for a period of time and conducted clinical evaluations with psychiatric and psychological interviews to detect PDs in either the community or clinical settings [13, 29, 51]. Our study was a register study of diagnosed PDs; hence, we cannot make a direct comparison to our findings regarding the “true” prevalence, because it is difficult to compare systematic assessments in samples with a large and complete clinical population. Indeed, in actual clinical practice, the diagnostic process may be guided by diagnostic interviews, but also, and more often clinical interviews. As such, we have no way to comment on the validity of the diagnoses reported. On the other hand, our aim was not to report scientifically valid diagnoses, but rather describe the actual practice of mental health practitioners in a European country.

In the present study, the most prevalent PD diagnoses were the unspecified PD diagnosis as well as the emotionally unstable, borderline type, which is consistent with prior findings for adult psychiatric services in Denmark [18, 44]. The trend toward the widespread diagnosis of unspecified PD is consistent with findings for adult patients in Danish Mental Services and may reflect that clinicians are either not truly interested in specifying further and/or that it is a useful diagnosis for referral [44], or that we lack developmentally appropriate diagnostic descriptions. Perhaps, and especially so in childhood and adolescence, it could be that a lack of developmentally sensitive diagnostic criteria makes it difficult for clinicians to specify their diagnosis beyond the general PD criteria. One reason for the high rates of borderline PD could be because the borderline PD population has a high rate of suicide attempts and self-injurious vis-à-vis, which often leads to contact with the psychiatric emergency departments and afterward referral to examination for PD [7, 22, 56]. Historically, there has been a general trend among clinicians to use the borderline diagnosis when a PD diagnosis is applied [50]. We found that the group of patients with PD had a significantly higher number of contacts with the psychiatric emergency departments compared to all other psychiatric disorders—at least up to 2013. Thus, even though PD patients are a relatively small group, they appear to use psychiatric emergency departments significantly more than patients with other psychiatric diagnoses, as also documented in other studies [7].

Taken together, our data suggest that, despite a rising trend in using PD diagnoses, PDs are still underdiagnosed among children and adolescents in Danish CAPS, at least when compared to prevalence rates from other research studies. Whereas caution is still warranted in children, there appears to exist a gap between research findings supporting the reliability, validity, and clinical utility of using PD diagnoses among adolescents vis-a-vis clinicians’ actual practice. The reluctance in clinical practice could also possibly account for the significant difference in the regional prevalence of PDs in Denmark. Although there are sociodemographic differences among the five regions, Denmark is a small and relatively homogenous country. Thus, we do not find it plausible that potential regional differences could account for the differences obtained in diagnostic practice. Part of the explanation for the regional differences is that Region Zealand Mental Health Services has a long tradition for clinical research on PDs [4, 31]. Through research, clinicians have been informed about the need for systematic evaluation of underlying personality pathology. On balance, however, the research focus on PDs in Region Zealand may also have contributed to overdiagnosis in this region, which is an important area for future research to address. In Denmark, patients need a referral from either GP, school psychologist or another doctor to be examined for psychiatric disorder in CAPS. Therefore, it is important that the group of people responsible for referrals be aware of the potential indicators of PD and refer the patient, so that they can obtain the correct diagnoses and treatment. It is important that those assessing referrals are competent in assessing PD pathology [48, 49]. Whatever the reasons, the finding of significant regional differences raises concerns, to the extent that we believe the differences are not due to actual regional differences but rather different clinical practices and diagnostic cultures, which suggests that children and adolescents are not being treated equally regardless of where they live.

The high quality and completeness of the Danish registers on which our data are based are a strength of the current study. Another strength of the study is that it uses real-world data, thereby reflecting nationwide actual clinical practice [39]. On the other hand, this also reflects a limitation considering that we have had no way to actually test and report on the reliability and validity of the diagnoses. The use of ICD-10 diagnoses may warrant caution when compared with studies using DSM-based diagnoses, yet research suggests that the DSM-IV and ICD-10 systems are largely concordant [42]. Furthermore, the development of pattern of co-occurrence and changes of psychiatric disorders over time have not been explored in this paper. This is an important area for future research to investigate further, to see how PDs are associated with internalizing and externalizing disorders over time.

Another limitation was the fact that due to the low prevalence of many specific PDs, we did calculate specific incidence and prevalence estimates for those. Some readers may wonder why dissocial PD was not included, the prevalence rate was very low. In the DSM system, use of this diagnosis before age 18 years is actually not endorsed, and though the ICD-10 are more allowing, it was not prevalent enough to present data for.

In conclusion, despite almost 3 decades of PD research demonstrating the reliability, validity, and clinical utility of PD diagnoses among children and adolescents, PD appears to be less frequent in Danish CAPS than in other international studies for reasons we cannot explain. Moreover, regional differences in the prevalence of PD diagnoses were also considerable, raising concerns about diagnostic practice as well as equal access to mental health care for children and adolescents with PD diagnoses. Apart from reluctance from clinicians and potential misconceptions about PD diagnosis legitimacy, at least in adolescence, it should also be recognized that in Denmark, although treatment is becoming increasingly available for this group of children and adolescents and their significant others, it is still not sufficient given the prevalence estimates reported in empirical studies. Thus, we would argue that widespread dissemination of treatments for children and adolescents with PDs would help facilitate wider recognition. On the other hand, if clinicians do not diagnose PDs but rather use other diagnoses, such as accentuated personality traits, mixed disorders of conduct and emotions, or even unspecified PD, the need for treatment may go undetected. As such, our findings have clinical implications, suggesting that apart from making treatments more available, perhaps the first form of early intervention needed is training and educating clinicians in CAPS about the importance and legitimacy of PD diagnoses in adolescents. That said, it must also be recognized that the use of PD diagnoses in childhood (i.e., before age 12 to 13) is still not easy nor empirically supported. Of course, lack of evidence is no evidence, and the lack of developmentally sensitive diagnostic criteria may make it even more difficult for clinicians. Such issues raise legitimate concerns about both under- and over-diagnosing PD in children. In adolescence, the research base are rather established, making concerns about stigma more a matter of educating clinicians than anything else. In childhood, however, evidence for PD diagnoses is lacking in terms of studies, and therefore, concerns here about over- or under-diagnosis are especially legitimate.

Future studies may track diagnostic practices both nationally, at the regional level, and globally to facilitate comparison and provide feedback for training clinicians and raising awareness. Also, the decision to remove any references to age in PD diagnostic criteria in the DSM-5 and ICD-11 warrants further empirical scrutiny to test if this actually improves diagnosis and treatment. Future research should also focus on marginalized groups, to see if they are over- or underdiagnosed. Moreover, future studies could focus on estimating trajectories and costs of PDs within the CAPS to facilitate informed decision-making regarding future organization and provision of services toward these children, adolescents, and their families.

### Supplementary Information

Below is the link to the electronic supplementary material.

Supplementary file1 (DOCX 15 KB)

Supplementary file2 (DOCX 16 KB)

Supplementary file3 (DOCX 25 KB)
