# Supplementary material for: Prevalence and incidence of personality disorders among children and adolescents in Danish mental health services: a nationwide register study
Source: Eur Child Adolesc Psychiatry. 2023 Aug 11;33(6):1731–40. doi: 10.1007/s00787-023-02274-w (PMC11211120; doi:10.1007/s00787-023-02274-w)
Supplement: Supplementary file 1 — Supplementary file1 (DOCX 15 KB) [file 787_2023_2274_MOESM1_ESM.docx]

**Supplemental Table S1: Number and proportion of personality disorder diagnoses compared to all psychiatric diagnoses in secondary Danish Child and Adolescent Psychiatric Services from 2007 to 2017.**

|  | **2007** | **2008** | **2009** | **2010** | **2011** | **2012** | **2013** | **2014** | **2015** | **2016** | **2017** |
| --- | --- | --- | --- | --- | --- | --- | --- | --- | --- | --- | --- |
| PD diagnoses (n) | 700 | 800 | 762 | 808 | 808 | 813 | 923 | 988 | 1046 | 952 | 851 |
| All psychiatric diagnoses (n) | 16.555 | 18.413 | 19.867 | 21.999 | 24.113 | 25.788 | 27.347 | 30.767 | 32.260 | 30.713 | 30.132 |
| % of PD diagnoses | 4,23 | 4,34 | 3,84 | 3,67 | 3,35 | 3,15 | 3,38 | 3,21 | 3,24 | 3,10 | 2,82 |

*Note.* PD = Personality disorder.
