# Supplementary material for: Prevalence and incidence of personality disorders among children and adolescents in Danish mental health services: a nationwide register study
Source: Eur Child Adolesc Psychiatry. 2023 Aug 11;33(6):1731–40. doi: 10.1007/s00787-023-02274-w (PMC11211120; doi:10.1007/s00787-023-02274-w)
Supplement: Supplementary file 2 — Supplementary file2 (DOCX 16 KB) [file 787_2023_2274_MOESM2_ESM.docx]

**Supplemental Table S2.** Incidence for personality disorder diagnoses as compared to all psychiatric diagnoses in secondary Danish child and adolescent psychiatric services from 2007 to 2017.

|  | Incidence of first psychiatric admission | | |  |
| --- | --- | --- | --- | --- |
|  | All psychiatric diagnoses | | PD diagnoses | |
|  | N |  | N | Incidence (%) |
| 2007 | 7865 |  | 318 | 4,04 |
| 2008 | 7777 |  | 309 | 3,97 |
| 2009 | 8133 |  | 298 | 3,66 |
| 2010 | 8206 |  | 260 | 3,17 |
| 2011 | 8321 |  | 228 | 2,74 |
| 2912 | 9106 |  | 259 | 2,84 |
| 2013 | 9438 |  | 279 | 2,96 |
| 2014 | 11762 |  | 318 | 2,70 |
| 2015 | 11957 |  | 308 | 2,58 |
| 2016 | 10786 |  | 240 | 2,23 |
| 2017 | 10716 |  | 195 | 1,82 |
| Mean | 9461 |  | 274 | 2,97 |

*Note.* PD = Personality disorder.
