# Supplementary material for: Prevalence and incidence of personality disorders among children and adolescents in Danish mental health services: a nationwide register study
Source: Eur Child Adolesc Psychiatry. 2023 Aug 11;33(6):1731–40. doi: 10.1007/s00787-023-02274-w (PMC11211120; doi:10.1007/s00787-023-02274-w)
Supplement: Supplementary file 3 — Supplementary file3 (DOCX 25 KB) [file 787_2023_2274_MOESM3_ESM.docx]

**Supplemental Table S3. Prevalence of personality disorder (PD) diagnoses stratified by age and year within secondary child and adolescent mental health services in Denmark.**

*Supplemental Table S3a. Prevalence of personality disorder (PD) diagnoses stratified by age in year 2007 within secondary child and adolescent mental services in Denmark.*

|  | PD | | All psychiatric diagnoses | | Prevalence of PD |
| --- | --- | --- | --- | --- | --- |
| Age in years | n | % | n | % | % |
| ≥10 | 20 | 2,86 | 7108 | 42,94 | 0,28 |
| 11-13 | 69 | 9,86 | 3539 | 21,38 | 1,95 |
| 14-15 | 235 | 33,57 | 3082 | 18,62 | 7,62 |
| 16-17 | 376 | 53,71 | 2826 | 17,07 | 13,31 |
| Total (N/prevalence) | 700 |  | 16.555 |  | 4,23 |

*Note.* PD = Personality disorder.

*Supplemental Table S3b. Prevalence of personality disorder (PD) diagnoses stratified by age in year 2008 within secondary child and adolescent mental services in Denmark.*

|  | PD | | All psychiatric diagnoses | | Prevalence of PD |
| --- | --- | --- | --- | --- | --- |
| Age in years | n | % | n | % | % |
| ≤10 | 31 | 3,88 | 8198 | 44,52 | 0,38 |
| 11-13 | 94 | 11,75 | 3956 | 21,48 | 2,38 |
| 14-15 | 306 | 38,25 | 3427 | 18,61 | 8,93 |
| 16-17 | 369 | 46,13 | 2832 | 15,38 | 13,03 |
| Total (N/prevalence) | 800 |  | 18.413 |  | 4,34 |

*Note.* PD = Personality disorder.

*Supplemental Table S3c. Prevalence of personality disorder (PD) diagnoses stratified by age in year 2009 within secondary child and adolescent mental services in Denmark.*

|  | PD | | All psychiatric diagnoses | | Prevalence of PD |
| --- | --- | --- | --- | --- | --- |
| Age in years | n | % | n | % | % |
| ≤10 | 33 | 4,33 | 9165 | 46,13 | 0,36 |
| 11-13 | 116 | 15,22 | 4339 | 21,84 | 2,67 |
| 14-15 | 330 | 43,31 | 3643 | 18,34 | 9,06 |
| 16-17 | 283 | 37,14 | 2720 | 13,69 | 10,40 |
| Total (N/prevalence) | 762 |  | 19.867 |  | 3,84 |

*Note.* PD = Personality disorder.

*Supplemental Table S3d. Prevalence of personality disorder (PD) diagnoses stratified by age in year 2010 within secondary child and adolescent mental services in Denmark.*

|  | PD | | All psychiatric diagnoses | | Prevalence of PD |
| --- | --- | --- | --- | --- | --- |
| Age in years | n | % | n | % | % |
| ≤10 | 30 | 3,71 | 10.448 | 47,49 | 0,29 |
| 11-13 | 144 | 17,82 | 4.907 | 22,31 | 2,93 |
| 14-15 | 337 | 41,71 | 3.851 | 17,51 | 8,75 |
| 16-17 | 297 | 36,76 | 2.793 | 12,70 | 10,63 |
| Total (N/prevalence) | 808 |  | 21.999 |  |  |

*Note.* PD = Personality disorder.

*Supplemental Table S3e. Prevalence of personality disorder (PD) diagnoses stratified by age in year 2011 within secondary child and adolescent mental services in Denmark.*

|  | PD | | All psychiatric diagnoses | | Prevalence of PD |
| --- | --- | --- | --- | --- | --- |
| Age in years | n | % | n | % | % |
| ≤10 | 36 | 4,66 | 11.756 | 48,75 | 0,31 |
| 11-13 | 165 | 20,42 | 5333 | 22,12 | 3,09 |
| 14-15 | 307 | 38,00 | 4000 | 16,59 | 7,68 |
| 16-17 | 300 | 37,13 | 3024 | 12,54 | 9,92 |
| Total (N/prevalence) | 808 |  | 24.113 |  | 3,35 |

*Note.* PD = Personality disorder.

*Supplemental Table S3f. Prevalence of personality disorder (PD) diagnoses stratified by age in year 2012 within secondary child and adolescent mental services in Denmark.*

|  | PD | | All psychiatric diagnoses | | Prevalence of PD |
| --- | --- | --- | --- | --- | --- |
| Age in years | n | % | n | % | % |
| ≤10 | 46 | 5,66 | 12.645 | 49,03 | 0,36 |
| 11-13 | 148 | 18,20 | 5556 | 21,54 | 2,66 |
| 14-15 | 304 | 37,39 | 4252 | 16,49 | 7,15 |
| 16-17 | 315 | 38,75 | 3335 | 12,93 | 9,45 |
| Total (N/prevalence) | 813 |  | 25.788 |  | 3,15 |

*Note.* PD = Personality disorder.

*Supplemental Table S3g. Prevalence of personality disorder (PD) diagnoses stratified by age in year 2013 within secondary child and adolescent mental services in Denmark.*

|  | PD | | All psychiatric diagnoses | | Prevalence of PD |
| --- | --- | --- | --- | --- | --- |
| Age in years | n | % | n | % | % |
| ≤10 | 72 | 7,80 | 13.579 | 49,65 | 0,53 |
| 11-13 | 182 | 19,72 | 5724 | 20,93 | 3,18 |
| 14-15 | 331 | 35,86 | 4406 | 16,11 | 7,51 |
| 16-17 | 338 | 36,62 | 3638 | 13,30 | 9,29 |
| Total (N/prevalence) | 923 |  | 27.347 |  | 3,38 |

*Note.* PD = Personality disorder.

*Supplemental Table S3h. Prevalence of personality disorder (PD) diagnoses stratified by age in year 2014 within secondary child and adolescent mental services in Denmark.*

|  | PD | | All psychiatric diagnoses | | Prevalence of PD |
| --- | --- | --- | --- | --- | --- |
| Age in years | n | % | n | % | % |
| ≤10 | 73 | 7,39 | 15.198 | 49,40 | 0,48 |
| 11-13 | 181 | 18,32 | 6226 | 20,24 | 2,91 |
| 14-15 | 340 | 34,41 | 5127 | 16,66 | 6,63 |
| 16-17 | 394 | 39,88 | 4216 | 13,70 | 9,35 |
| Total (N/prevalence) | 988 |  | 30.767 |  | 3,21 |

*Note.* PD = Personality disorder.

*Supplemental Table S3i. Prevalence of personality disorder (PD) diagnoses stratified by age in year 2015 within secondary child and adolescent mental services in Denmark.*

|  | PD | | All psychiatric diagnoses | | Prevalence of PD |
| --- | --- | --- | --- | --- | --- |
| Age in years | n | % | n | % | % |
| ≤10 | 87 | 8,32 | 16.092 | 49,88 | 0,54 |
| 11-13 | 168 | 16,06 | 6365 | 19,73 | 2,64 |
| 14-15 | 379 | 36,23 | 5430 | 16,83 | 6,98 |
| 16-17 | 412 | 39,39 | 4375 | 13,56 | 9,42 |
| Total (N/prevalence) | 1046 |  | 32.262 |  | 3,24 |

*Note.* PD = Personality disorder.

*Supplemental Table S3j. Prevalence of personality disorder (PD) diagnoses stratified by age in year 2016 within secondary child and adolescent mental services in Denmark.*

|  | PD | | All psychiatric diagnoses | | Prevalence of PD |
| --- | --- | --- | --- | --- | --- |
| Age in years | n | % | n | % | % |
| ≤10 | 89 | 9,35 | 15.545 | 50,61 | 0,57 |
| 11-13 | 161 | 16,91 | 6102 | 19,87 | 2,64 |
| 14-15 | 359 | 37,71 | 5110 | 16,64 | 7,03 |
| 16-17 | 343 | 36,03 | 3957 | 12,88 | 8,67 |
| Total (N/prevalence) | 952 |  | 30.714 |  | 3,10 |

*Note.* PD = Personality disorder.

*Supplemental Table S3k. Prevalence of personality disorder (PD) diagnoses stratified by age in year 2017 within secondary child and adolescent mental services in Denmark.*

|  | PD | | All psychiatric diagnoses | | Prevalence of PD |
| --- | --- | --- | --- | --- | --- |
| Age in years | n | % | n | % | % |
| ≤10 | 73 | 8,58 | 14.953 | 49,62 | 0,49 |
| 11-13 | 168 | 19,74 | 6193 | 20,55 | 2,71 |
| 14-15 | 305 | 35,84 | 5109 | 16,95 | 5,97 |
| 16-17 | 305 | 35,84 | 3880 | 12,88 | 7,86 |
| Total (N/prevalence) | 851 |  | 30.135 |  | 2,82 |

*Note.* PD = Personality disorder.
